# Predator Cat Odors Activate Sexual Arousal Pathways in Brains of *Toxoplasma gondii* Infected Rats

**DOI:** 10.1371/journal.pone.0023277

**Published:** 2011-08-17

**Authors:** Patrick K. House, Ajai Vyas, Robert Sapolsky

**Affiliations:** 1 Program in Neuroscience, Stanford University, Stanford, California, United States of America; 2 School of Biological Science, Nanyang Technology University, Singapore; 3 Departments of Biology, Neurology and Neurological Sciences, and of Neurosurgery, Stanford University, Stanford, California, United States of America; Université Pierre et Marie Curie, France

## Abstract

Cat odors induce rapid, innate and stereotyped defensive behaviors in rats at first exposure, a presumed response to the evolutionary pressures of predation. Bizarrely, rats infected with the brain parasite *Toxoplasma gondii* approach the cat odors they typically avoid. Since the protozoan *Toxoplasma* requires the cat to sexually reproduce, this change in host behavior is thought to be a remarkable example of a parasite manipulating a mammalian host for its own benefit. *Toxoplasma* does not influence host response to non-feline predator odor nor does it alter behavior on olfactory, social, fear or anxiety tests, arguing for specific manipulation in the processing of cat odor. We report that *Toxoplasma* infection alters neural activity in limbic brain areas necessary for innate defensive behavior in response to cat odor. Moreover, *Toxoplasma* increases activity in nearby limbic regions of sexual attraction when the rat is exposed to cat urine, compelling evidence that *Toxoplasma* overwhelms the innate fear response by causing, in its stead, a type of sexual attraction to the normally aversive cat odor.

## Introduction

A fascinating phenomenon in behavioral biology is the ability of parasites to manipulate host behavior for their own benefit. A handful of examples are noted for insect [1], [2] and crustacean [3] hosts, but rarely so in mammals. The extraordinary effectiveness of the mammalian blood brain barrier denies most pathogens access to the privileged central nervous system, the seat of will.

*Toxoplasma gondii* is an obligate, single-celled protozoan parasite capable of crossing into the central nervous system of any warm-blooded vertebrate. *Toxoplasma* requires the cat intestine to reproduce sexually, is shed in cat feces, and must make its way from the ground to another cat host [4].

*Toxoplasma* manages this in part by infecting ground-dwelling rats who, remarkably, begin selectively preferring areas with cat urine [5], [6], [7]. Infected rats retain normal defensive behavior to non-feline predator odor and normal performance on memory, anxiety, fear and social tasks [6], [8]. This specific preference for cat odor is likely an adaptive manipulation by *Toxoplasma*, increasing infected rat predation rates and facilitating *Toxoplasma* transmission to the cat.

Little is known about how *Toxoplasma* inspires this manipulation. By two weeks post infection, *Toxoplasma* has settled throughout the host rat brain in spherical cysts approximately 50–70 µm in diameter [4]. Intriguingly, cysts show a slight preference for limbic system regions responsive to both predator stimuli and sexual stimuli, regions responsible for gating innate approach and avoidance behaviors [6]. We investigated the effect of *Toxoplasma* on the neural activity in limbic system regions involved in both ‘defensive’ and ‘reproductive’ innate behavior. Neural activity was quantified using the immediate early gene c-Fos, a proxy for neural activity (see **Materials and Methods**).

## Results

**Figure 1:**
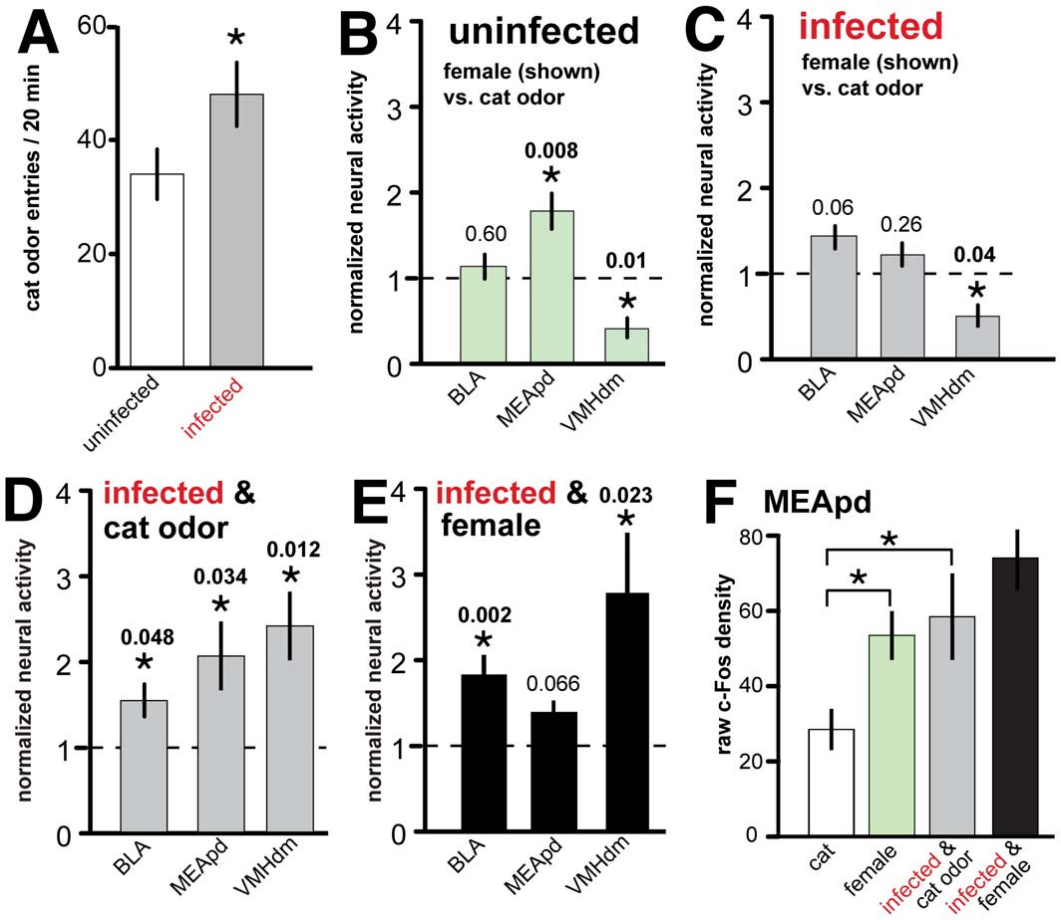
*Toxoplasma* Infection Alters the Limbic Response in Rats Exposed to Cat Odor. (A) *Toxoplasma* infected rats spent more time exploring cat urine than uninfected rats. (B) In uninfected animals, female odor induces relatively greater ‘reproductive’ MEApd activity and relatively diminished ‘defensive’ VMHdm activity, as expected. (C) In infected animals, activity levels in the MEApd are the same, regardless of whether exposed to a ‘reproductive’ or ‘defensive’ odor. (D) Neural activity in infected male rats, normalized to uninfected controls, after exposure to cat urine. Infection increased neural activity in the BLA, MEApd and VMHdm. (E) Neural activity in infected male rats, normalized to uninfected controls, after exposure to an estrous female. Infection again increased neural activity in the BLA and VMHdm, but did not change MEApd levels. (F) *Toxoplasma* infection increased ‘reproductive’ MEApd neural activity during exposure to cat odor, mimicking neural activity in uninfected animals exposed to a female rat. *MEApd*, posterodorsal medial amygdala; *VMHdm*, ventromedial hypothalamus, dorsomedial part; *BLA*, basolateral amygdala. *P* values listed where appropriate.

We first confirmed limbic activity during exposure to either cat urine or an inaccessible estrous female. As expected [9], [10], [11] in uninfected rats, cat urine increased neural activity in the ‘defensive’ ventromedial hypothalamus, dorsomedial part (VMHdm) (**Figure 1B** and **Table S1**). Exposure to an estrous female rat increased activity in the ‘reproductive’ posterodorsal medial amygdala (MEApd) (**Figure 1B** and **Table S1**) [11].

*Toxoplasma* infection made rats spend more time exploring cat urine (**Figure 1A**) and increased neural activity in the MEApd, VMHdm and basolateral amygdala (BLA) (**Figure 1C and 1D**) during this exploration. During exposure to an estrous female, *Toxoplasma* increased activity in the VMHdm and BLA, but did not significantly alter MEApd activity (**Figure 1E**) relative to uninfected controls. *Toxoplasma* infected rats had reduced volumes of both the MEApd and the posteroventral medial amygdala (MEApv) (**Figure S1** and see **Discussion S1**).

## Discussion

**Figure 2:**
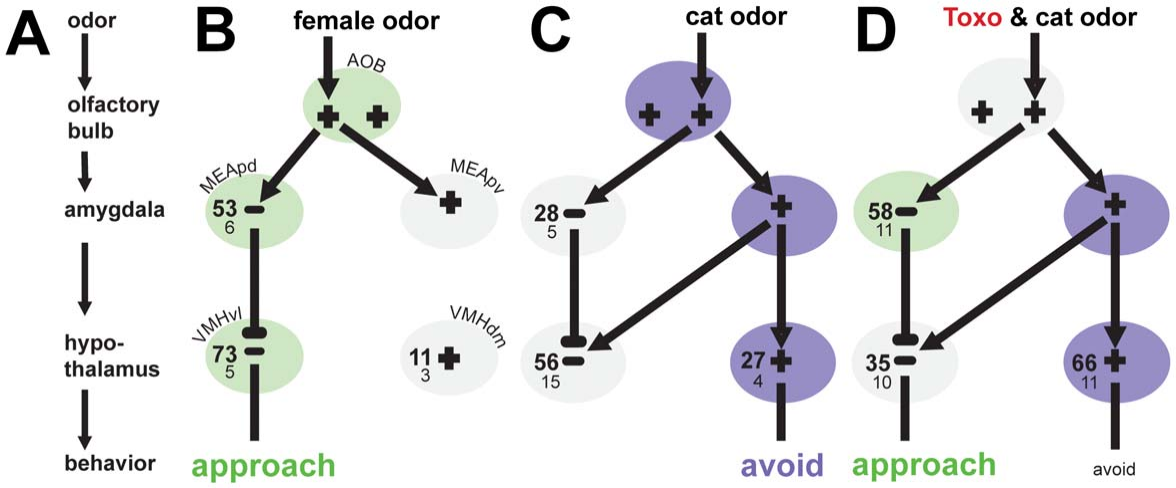
Schematic Model of Toxoplasma Induced Changes to Host Limbic System. (A) General schematic model of limbic brain activity in a male rat after exposure to an emotionally salient odor. (B) Exposure to an inaccessible estrous female activates the ‘reproductive’ pathway, producing robust MEApd activity and evoking approach behavior. (C) Exposure to cat urine activates the ‘defensive’ pathway, producing robust activation of excitatory (+) VMHdm neurons and evoking defensive, aversion behavior. Excitatory (pointed arrow) projections from the MEApv activate inhibitory (−) VMHvl neurons, countering inhibitory (blunted arrow) projections from the MEApd and suppressing any approach behavior. (D) Proposed model for *Toxoplasma* rats during exposure to cat urine. Robust MEApd activity biases toward approach behavior. Aversion behavior remains, but is lessened. Raw density of c-Fos (large print) ±SEM (small print) activity is given for each region during relevant odor exposure. Model adapted from Choi 2005. *AOB*, accessory olfactory bulb; *MEA*, medial amygdala; *VMH*, ventromedial hypothalamus.

Rats have separate ‘defensive’ and ‘reproductive’ pathways gating innate behavioral response to, respectively, predator or sexual stimuli [11]. Given the precipitous evolutionary pressures of both reproduction and predation, these pathways run as direct projections from the olfactory bulb to the limbic system and generate rapid and stereotyped behavioral output (see **Figure 2B** and **Figure 2C**). Although functionally distinct, the limbic ‘defensive’ and ‘reproductive’ pathways run in parallel through the medial amygdala and hypothalamus in close anatomical proximity. Previous findings of increased *Toxoplasma* cyst density in these areas compel the possibility that *Toxoplasma* is somehow perturbing its surrounding neural environment and thereby manipulating the host response to cat urine. We find, indeed, that *Toxoplasma* infection perturbs the ‘defensive’ pathway in the infected rat during exposure to cat urine, shifting neural activity to the nearby ‘reproductive’ pathway, specifically the MEApd (see **Figure 2D**).

The MEApd projects robustly to hypothalamic nuclei involved in sexual arousal and the generation of approach behavior. The MEApd is responsive to a variety of social odorants [12], but responds strongest to opposite-sex mating stimuli. Lesioning the MEApd reduces, specifically, attraction to opposite-sex odors [13]. Interestingly, *Toxoplasma* increased MEApd activity during cat urine exposure to levels mimicking uninfected rats during female exposure (**Figure 1F**). This suggests that the specifically increased magnitude of MEApd activity in male *Toxoplasma* infected rats is biasing the processing of the cat urine toward the sexual, ‘reproductive’ pathway (**Figure 2D**). Plausibly, this shift is altering the salience of the cat urine stimuli and mitigating the defensive response by creating, in its stead, a competing attraction to the cat urine.

Little is known about how, if at all, *Toxoplasma* cysts exert themselves in the host brain. Much work remains to be done, based on striking findings that *Toxoplasma* raises whole brain dopamine levels in mice by up to 15% [14] and that dopamine receptor antagonists block rodent host attraction to cat urine [7]. These data suggest a link between dopamine, a primary neurotransmitter in decision-making and reward, and the altered behavior. Intriguingly, the *Toxoplasma* genome contains a homolog of tyrosine hydroxylase [15], the rate-limiting enzyme in the vertebrate synthesis of dopamine, raising the possibility that *Toxoplasma* is altering dopamine levels by synthesizing its own tyrosine hydroxylase.

From the ground, *Toxoplasma* finds its way into other hosts besides rats, including cows, sheep, pigs and many grazing livestock. Ingestion of undercooked meat from infected livestock and the profligacy of private cat ownership are responsible for a strikingly high number of human chronic *Toxoplasma* infections. Approximately one-third of humans are seropositive for *Toxoplasma* across the world [16], and several recent studies find infection increases risk for schizophrenia [17], [18], [19] and obsessive compulsive disorder [20], diseases noted for elevated dopamine levels and disturbed amygdala function [21]. Our results are therefore of wide interest, as the ability of *Toxoplasma* to dramatically alter host behavior and proper amygdala functioning may extend beyond the rat into ancillary *Toxoplasma* hosts, including humans.

## Materials and Methods

### Ethics Statement

The use and care of animals complied with the guidelines of the Animal Advisory Committee at Stanford University. The protocol was approved by the Institutional Animal Care and Use Committee (Protocol #: APLAC-11603).

### Experimental Design

Animals were split into four groups: cat-urine uninfected (*n* = 9), female-odor uninfected (*n* = 9), cat-urine infected (n = 9) or female-odor infected (*n* = 9). On the day of sacrifice, animals were exposed to either cat odor or an inaccessible estrous female. Brains were collected and regions of interest were analyzed for c-Fos activation. For stereological analysis, twelve animals were split into two groups: stereology-control (n = 6) and stereology-infected (n = 6).

### Animals

All studies involved male Long Evans rats. The animals were housed in groups of three, kept on a 12 hr light/dark cycle and given food and water *ad libitum*. Behavioral testing occurred during the light cycle.

### Toxoplasma injection

The *Toxoplasma* infected groups (*n* = 18 for cat-urine infected groups, *n* = 6 for stereolgy-infected group) were injected i.p. with approximately 10∧7 *Toxoplasma* tachyzoites. We employed a Prugnaud strain of *Toxoplasma*, maintained as tachyzoites by passage in human foreskin fibroblast monolayers. Infected fibroblasts were syringe lysed using a 27-gauge needle and injected into animals. Animals were either infected i.p. with *Toxoplasma* tachyzoites or mock-infected with sterile PBS. Behavior experiments and c-Fos quantification was performed six weeks post-infection.

### Odor Exposure and Behavior

For cat-urine groups, a towel with 1 ml bobcat urine was clipped to a rack above the home cage for 20 min. For female-odor groups, an inaccessible estrous female was placed in the home cage for 20 min, separated from the male rats by a plastic divider with holes in it. Male rats could not touch the female. Video recordings were scored by A.V. Briefly, in the videos *post hoc* ‘incentive zones’ were created around the feline urine or the towel and the number of nose pokes into this area were scored across the twenty minute period.

### Tissue Fixation

Animals were sacrificed 90 min after the end of the 20 minute exposure to either cat odor or a female rat. Animals were deeply anaesthetized and transcardially perfused with 4% paraformaldehyde (PFA) made in 0.1 M phosphate buffer (PB). The brains were removed from the skull and postfixed in 4% PFA overnight. Blocks containing the amygdala and hypothalamus were cut on a cryostat and subsequently sectioned into 40 µm thick sections.

### Immunohistochemistry

Sections were incubated in 1% H202 for 15 min and then incubated for 90 hrs at 4°C with a c-Fos primary antibody (1∶2000, sc52 Rabbit Polyclonal, Santa Cruz Biotechnology) diluted in PBS^+^ (0.1 M PBS with 0.2% Triton-X and 0.1% BSA). Sections were then incubated for 1 hr in a secondary antibody solution (1∶400, biotinylated anti-rabbit IgG, Vector Laboratories), followed by incubation for 1 hr in Vectastain Elite ABC Reagent (1∶25, Vectastain Elite ABC Kit, PK6101 Rabbit, Vector Laboratories). Next, sections were incubated for 6 min in a DAB solution (DAB Substrate Kit SK-4100, Vector Laboratories). Sections were mounted and coverslipped. Sections were washed in 0.1 M PBS for 30 min between each of these steps and all steps were done under agitation.

### c-Fos Counting

For technical reasons, not all brains could be counted. Two animals were lost in the cat-urine uninfected group (leaving *n* = 7), one in the female-odor uninfected group (leaving *n* = 8) and one in the cat-urine *Toxoplasma* group (leaving *n* = 8). Regions of interest were traced in Stereo Investigator software and scored. Only darkly-labeled oval shaped nuclei were counted as c-Fos positive. The area of the region of interest was scored using the Cavalieri Estimator tool in Stereo Investigator software. The number of positive nuclei was divided by the area of the region to arrive at c-Fos density per region of interest.

### Stereology

A systematic and randomly sampled series of sections through regions of interest was used to estimate volumes. Specifically, 40 µm coronal sections throughout the entire region of interest were cut and cresyl-violet stained. The area of the ROI in every fourth section was estimated using the Cavalieri Estimator tool in Stereo Investigator software. The first section in the series was randomly selected from among the first four sections. The distance between the upper surfaces of the sections was 160 µm (4×40 µm). Areas were recorded for each ROI as described above and total volumes were calculated using the Stereo Investigator software.

### Statistical Analysis

Behavior was analyzed using one-way analysis of variance (ANOVA) to compare between uninfected and *Toxoplasma* infected groups exposed to cat urine. Values are reported as mean ± SEM throughout. For c-Fos counts and volume data, an independent-samples T test was conducted. A *P* value of <0.05 indicates statistical significance throughout.

## Supporting Information

Figure S1 **Toxoplasma Alters Volumes and c-Fos Expression of Limbic Regions Involved in Processing Cat Odor.** (A) Schematic diagram (adapted from Paxinos and Watson 2007) of coronal slices of rat brain showing areas of c-Fos and volume quantification. (B) Volumes of amygdalar and hypothalamic regions of interest in uninfected and *Toxoplasma*-infected animals. Coronal 40 um sections were taken throughout the whole region and 3-dimensional volumes were calculated via stereological analysis. (C) Digital camera lucida drawings of c-Fos signal in the lateral amygdala in uninfected (left) or infected (right) rats. (D) Digital camera lucida drawings of c-Fos signal in the medial amygdala in uninfected (left) or infected (right) rats. (E) c-Fos photomicrograph from which (C) is based. (F) c-Fos photomicrograph from which (D) is based.
(TIF)

Table S1 Density of c-Fos positive cells (mean ± SEM).
(PDF)

Discussion S1 (DOC)
