# Supplementary figures and images for: Predator Cat Odors Activate Sexual Arousal Pathways in Brains of Toxoplasma gondii Infected Rats
Source: PLoS One. 2011 Aug 17;6(8):e23277. doi: 10.1371/journal.pone.0023277 (PMC3157360; doi:10.1371/journal.pone.0023277)

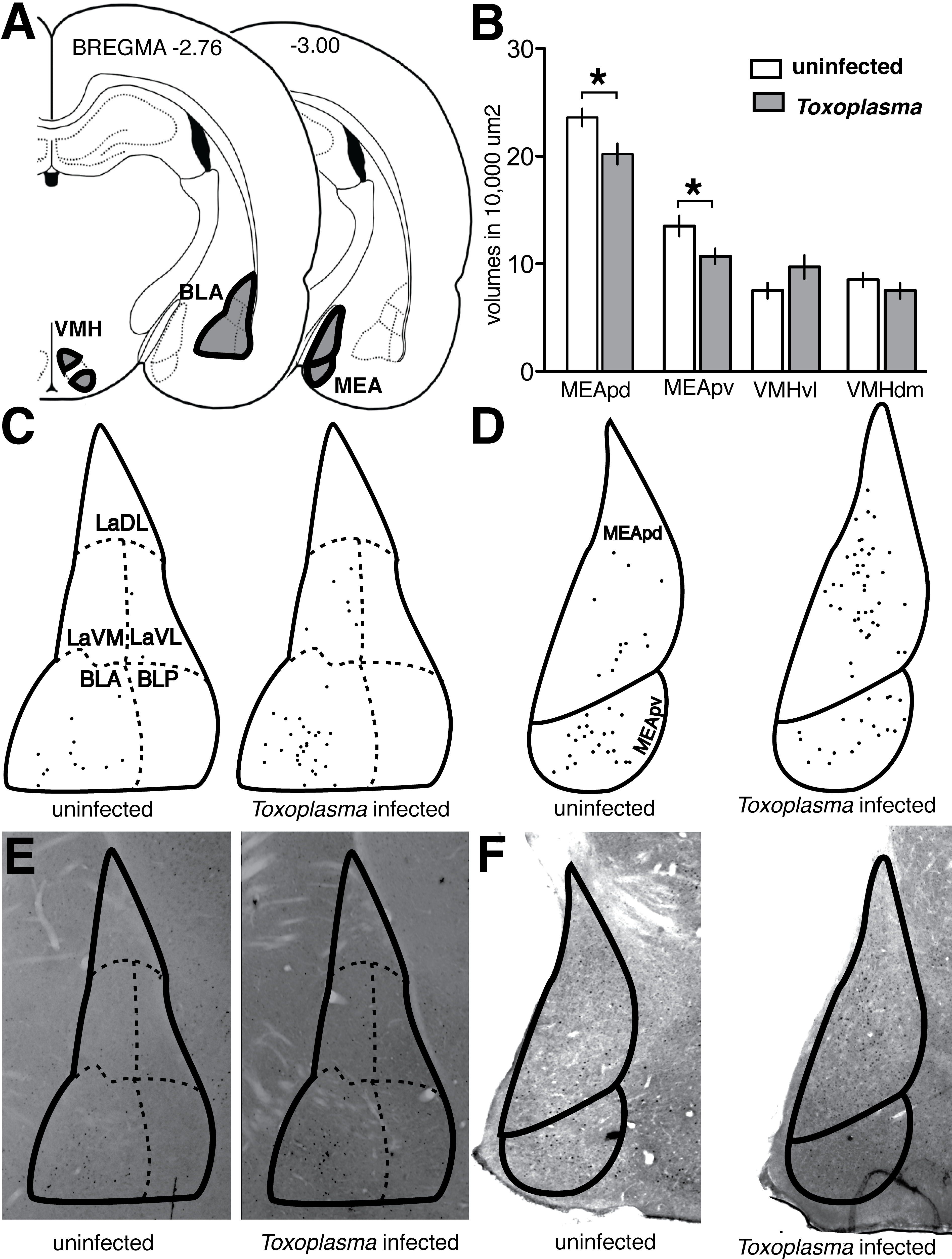

Supplement: Figure S1 — Toxoplasma Alters Volumes and c-Fos Expression of Limbic Regions Involved in Processing Cat Odor. (A) Schematic diagram (adapted from Paxinos and Watson 2007) of coronal slices of rat brain showing areas of c-Fos and volume quantification. (B) Volumes of amygdalar and hypothalamic regions of interest in uninfected and Toxoplasma-infected animals. Coronal 40 um sections were taken throughout the whole region and 3-dimensional volumes were calculated via stereological analysis. (C) Digital camera lucida drawings of c-Fos signal in the lateral amygdala in uninfected (left) or infected (right) rats. (D) Digital camera lucida drawings of c-Fos signal in the medial amygdala in uninfected (left) or infected (right) rats. (E) c-Fos photomicrograph from which (C) is based. (F) c-Fos photomicrograph from which (D) is based. (TIF) [file pone.0023277.s001.tif]
