# Supplementary material for: Predator Cat Odors Activate Sexual Arousal Pathways in Brains of Toxoplasma gondii Infected Rats
Source: PLoS One. 2011 Aug 17;6(8):e23277. doi: 10.1371/journal.pone.0023277 (PMC3157360; doi:10.1371/journal.pone.0023277)
Supplement: Table S1 — Density of c-Fos positive cells (mean ± SEM). (PDF) [file pone.0023277.s002.pdf]

Region	Cat odor<br>Uninfected<br>(*n = 9)*	Female odor<br>Uninfected<br>(*n = 9)*	Cat odor<br>*Toxoplasma*<br>(*n* = 9)	Female Odor<br>*Toxoplasma*<br>(n = 9)
Lateral Amygdala				
1. LAdl (dorsolateral)	0 ± 0	1 ± 1	6 ± 2 *	4 ± 3
2. LAvm (ventromedial)	15 ± 7	22 ± 6	15 ± 6	41 ± 8 #
3. LAvl (ventrolateral)	3 ± 1	10 ± 3 *	0 ± 0	20 ± 7
Basolateral Amygdala				
4. BLA (anterior)	41 ± 2	39 ± 5	54 ± 6 *	74 ± 8 #
5. BLP (posterior)	5 ± 2	5 ± 2	4 ± 2	6 ± 2
Medial amygdaloid nucleus				
6. MEApd (posterodorsal)	28 ± 5	53 ± 6 *	58 ± 11 *	74 ± 8
7. MEApv (posteroventral)	57 ± 10	43 ± 8	57 ± 11	51 ± 12
Ventromedial hypothalamic nucleus				
8. VMHdm (dorsomedial)	27 ± 4	11 ± 3 *	66 ± 11 *	34 ± 8 #
9. VMHvl (ventrolateral)	56 ± 15	73 ± 5	35 ± 10	54 ± 10

Independent-samples T Test: * indicates $P < 0.05$ relative to Cat Odor Uninfected group; # indicates P < .0.05 relative to Female Odor Uninfected group
