# Supplementary material for: Predator Cat Odors Activate Sexual Arousal Pathways in Brains of Toxoplasma gondii Infected Rats
Source: PLoS One. 2011 Aug 17;6(8):e23277. doi: 10.1371/journal.pone.0023277 (PMC3157360; doi:10.1371/journal.pone.0023277)
Supplement: Discussion S1 — (DOC) [file pone.0023277.s003.doc]

**SUPPORTING DISCUSSION**

Somewhat surprisingly, uninfected animals exposed to cat urine show activation in the MEApd and BLA (**Table S1**). Previous studies investigating predator odor exposure in rats find no activation in either the MEApd or BLA [1,2]; however, these studies expose rats to either a collar worn by a cat or a towel rubbed on a cat. Our study is, to the best of our knowledge, the first investigation of c-Fos activity in rats exposed to cat urine. In natural environments, cat urine is frequently an indirect marker of feline proximity, likely to be further temporally and spatially removed from an actual cat than cat fur, which the towel and collar effectively imitate.

Behaviorally, rats show less aversion and less defensive activity in response to cat urine than to cat towel or cat collar. Moreover, rats cannot be conditioned using cat urine as the aversive stimuli, but will successfully condition context to cat collar or cat towel. Thus, different types of cat predator stimuli evoke different behaviors, depending on the relative salience of the stimuli, and our findings show these differences reflected in the c-Fos activation of regions such as the MEApd and BLA in response to cat urine.

Infection reduced both MEApd and MEApv volumes in rats (**Figure S1B**), further evidence that *Toxoplasma* infection disturbs the host limbic system. *Toxoplasma* may be blunting the ability of the “defensive” pathway to suppress the “reproductive” pathway. The MEApv is thought to be in a privileged position to gate ambiguous stimuli and limbic output [1] given its activation by either reproductive or defensive stimuli and its opposing projections to both the reproductive and defensive regions of the VMH.

The MEA receives strong intra-amygdalar afferents from the lateral and accessory basal nuclei, components of the BLA thought to regulate emotional arousal and salience [3,4] Plausibly, BLA hyperactivation in *Toxoplasma-*infected rats (**Table S1**) is influencing downstream MEA circuitry.

**SUPPORTING REFERENCES**

1. Choi GB, Dong H-W, Murphy AJ, Valenzuela DM, Yancopoulos GD, et al. (2005) Lhx6 delineates a pathway mediating innate reproductive behaviors from the amygdala to the hypothalamus. *Neuron* 46: 647-660.

2. Dielenberg RA, Hunt GE, McGregor IS (2001) "When a rat smells a cat": the distribution of Fos immunoreactivity in rat brain following exposure to a predatory odor. *Neuroscience* 104: 1085-1097.

3. Takahashi LK, Hubbard DT, Lee I, Dar Y, Sipes SM (2007) Predator odor-induced conditioned fear involves the basolateral and medial amygdala. *Behav Neurosci* 121: 100-110.

4. Pitkänen A, Jolkkonen E, Kemppainen S (2000) Anatomic heterogeneity of the rat amygdaloid complex. *Folia Morphol* (Warsz) 59: 1-23.
